# Supplementary material for: The causal role of circulating inflammatory markers in osteoporosis: a bidirectional Mendelian randomized study
Source: Front Immunol. 2024 Jul 18;15:1412298. doi: 10.3389/fimmu.2024.1412298 (PMC11291241; doi:10.3389/fimmu.2024.1412298)
Supplement: Supplementary file 1 [file DataSheet_1.zip › supplementary material/OSTEOPOROSIS-CIM/GCST90274758.tsv.gz_forest.pdf]

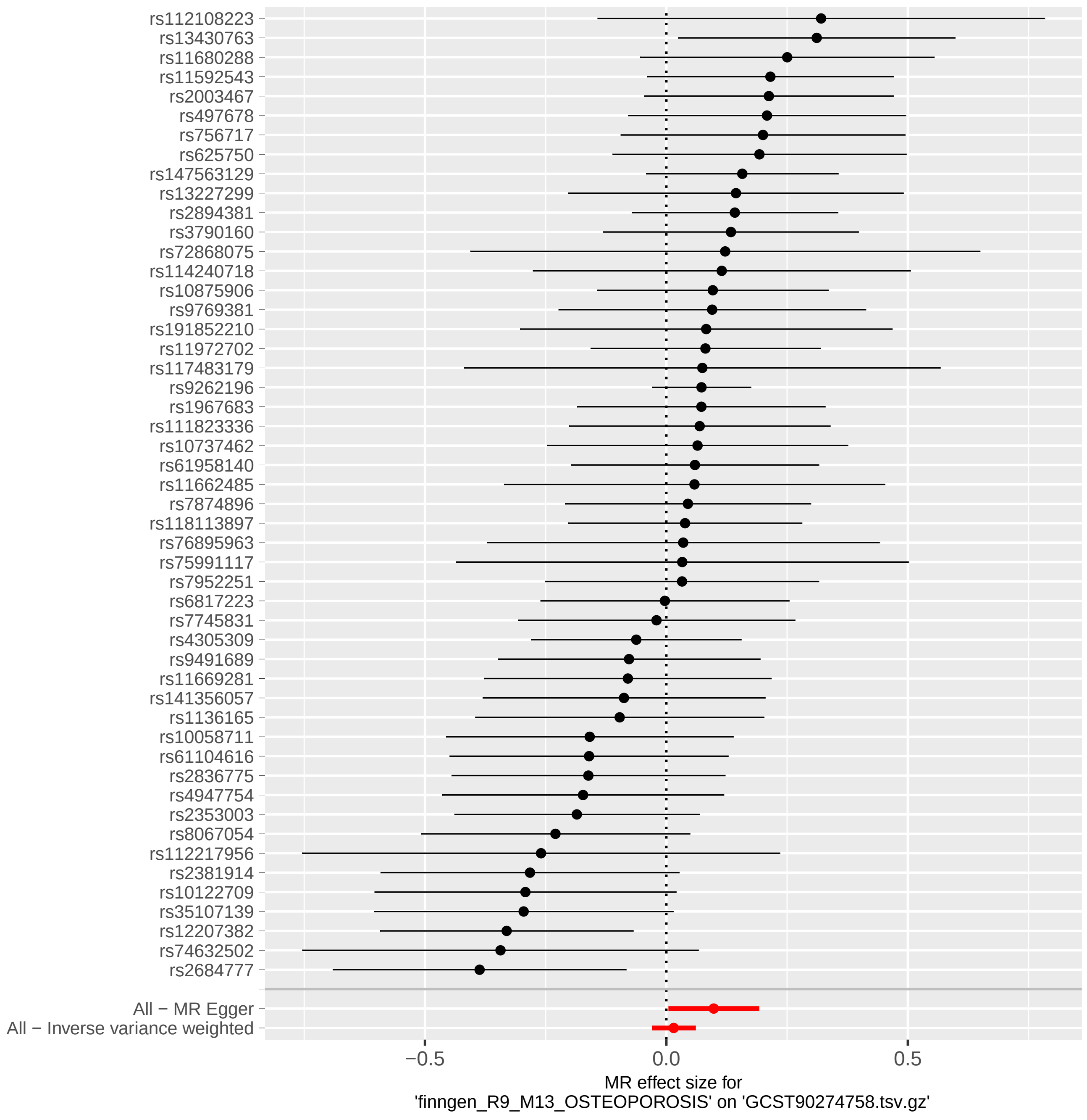
rs112108223
rs13430763
rs11680288
rs11592543
rs2003467
rs497678
rs756717
rs625750
rs147563129
rs13227299
rs2894381
rs3790160
rs72868075
rs114240718
rs10875906
rs9769381
rs191852210
rs11972702
rs117483179
rs9262196
rs1967683
rs111823336
rs10737462
rs61958140
rs11662485
rs7874896
rs118113897
rs76895963
rs75991117
rs7952251
rs6817223
rs7745831
rs4305309
rs9491689
rs11669281
rs141356057
rs1136165
rs10058711
rs61104616
rs2836775
rs4947754
rs2353003
rs8067054
rs112217956
rs2381914
rs10122709
rs35107139
rs12207382
rs74632502
rs2684777
All − MR Egger
All − Inverse variance weighted
−0.5
0.0
0.5
MR effect size for
'finngen_R9_M13_OSTEOPOROSIS' on 'GCST90274758.tsv.gz'
